# Improved therapeutic efficacy of unmodified anti-tumor antibodies by immune checkpoint blockade and kinase targeted therapy in mouse models of melanoma

**DOI:** 10.18632/oncotarget.27868

**Published:** 2021-01-19

**Authors:** Rolando Pérez-Lorenzo, Stephanie O. Erjavec, Angela M. Christiano, Raphael Clynes

**Affiliations:** ^1^Department of Dermatology, Columbia University, New York, NY 10032, USA; ^2^Department of Genetics and Development, Columbia University, New York, NY 10032, USA; ^3^Department of Medicine, Columbia University, New York, NY 10032, USA

**Keywords:** anti-tumor antibodies, targeted therapy, immunotherapy, combination therapies, melanoma

## Abstract

The use of specific anti-tumor antibodies has transformed the solid cancer therapeutics landscape with the relative successes of therapies such as anti-HER2 in breast cancer, and anti-EGFR in HNSCC and colorectal cancer. However, these therapies result in toxicity and the emergence of resistant tumors. Here, we showed that removing immune suppression and enhancing stimulatory signals increased the anti-tumor activity of unmodified TA99 antibodies (anti-TYRP1) with a significant reduction of growth of solid tumors and lung metastases in mouse models of melanoma. Immune checkpoint blockade enhanced the efficacy of TA99, which was associated with greater CD8^+^/Foxp3^+^, NK1.1^+^ and dendritic cell infiltrates, suggestive of an increased anti-tumor innate and adaptive immune responses. Further, MEK inhibition in melanoma cell lines increased the expression of melanosomal antigens *in vitro*, and combining TA99 and MEKi *in vivo* resulted in enhanced tumor control. Moreover, we found an improved therapeutic effect when YUMM tumor-bearing mice were treated with TA99 combined with MEKi and immune checkpoint blockade (anti-PD1 and anti-CTLA4). Our findings suggest that MEKi induced an increased expression of tumor-associated antigens, which in combination with anti-tumor antibodies, generated a robust adaptive anti-tumor response that was sustained by immune checkpoint inhibition therapy. We postulate that combining anti-tumor antibodies with standard-of-care strategies such as immune checkpoint blockade or targeted therapy, will improve therapeutic outcomes in cancer.

## INTRODUCTION

It is well accepted that tumor development and progression is usually controlled by immunosurveillance mechanisms in which specific and non-specific immunological responses are constantly mounted against tumor cells [1]. Growing evidence points toward a correlation between high immunogenicity and immune responsiveness of tumors [2]. In melanoma, this concept is supported by findings such as spontaneous remissions, the existence of metastatic tumors without identifiable primary lesions, and the presence of infiltrating T lymphocytes capable of recognizing melanoma-derived antigens in primary tumors and metastatic lesions [3, 4].

The treatment of certain cancers has been transformed by the use of specific anti-tumor antibody therapeutics, including FDA-approved monoclonal antibodies for the treatment of breast, colon [5], head and neck [6], multiple myeloma [7], chronic lymphocytic leukemia [8], and non-Hodgkin’s lymphoma [9]. Nevertheless, clinical remissions are infrequent and transient in advanced stage solid tumors treated with anti-tumor antibodies, even with concomitant chemotherapy [10, 11]. Early clinical successes with anti-tumor antibody therapy (anti-CD20, anti-HER2, anti-EGFR) were exclusively attributed to the interruption of their respective signaling pathways, but recent evidence suggests an essential role for innate as well as adaptive immunity in the therapeutic outcome.

Passive administration of anti-tumor antibodies generally functions by targeting malignant cells through IgG-mediated antibody-dependent cellular cytotoxicity (ADCC), which is a rapid but relatively short-acting anti-tumor response. Alternatively, we and others also demonstrated that the administration of anti-tumor antibodies induces long-lasting FcR-dependent tumor specific immunity in the host, with kinetics consistent with an induced adaptive immune response against the tumor [12, 13]. In this model, anti-tumor antibodies, alone or in combination with chemotherapy, will promote innate cell-mediated ADCC (e.g., macrophages), and the capture and processing of antigens by antigen presenting cells (APC), with the subsequent stimulation and homing of antigen-specific effector T lymphocytes to the tumor site, leading to tumor elimination, a phenomenon we and others referred to as the “vaccinal effect” [12–14].

However, the activation of adaptive immune responses is also strictly regulated *in vivo* by inhibitory signaling pathways, which can be hijacked by successful tumor cells as an immune evasion mechanism. In order to overcome these regulatory mechanisms, different therapeutic strategies using antibody-mediated immune checkpoint blockade (ICB; i.e., anti-CTLA4 and anti-PD1) have had a profound impact in anti-cancer therapy by improving survival [15].

Here, we investigated the potential of currently available targeted therapies and ICB to enhance the therapeutic efficacy of unmodified anti-tumor antibodies. With the use of the B16 and YUMM mouse models of melanoma and the anti-TYRP1 mouse monoclonal antibody TA99, we demonstrated that the therapeutic effects of these unmodified anti-tumor antibodies can be enhanced by ICB (anti-PD1 and anti-CTLA4 monoclonal antibodies) through the stimulation of both innate and adaptive anti-tumor immune responses. In addition, we found that the MEK inhibitor (trametinib)-induced increased expression of melanosomal antigens further enhanced the anti-melanoma response to combination therapy with anti-tumor antibodies and immune checkpoint blockade in mouse models of melanoma.

## RESULTS

### Elimination of T regulatory cells enhances the efficacy of TA99 antibodies in the B16 mouse melanoma model

**Figure 1:**
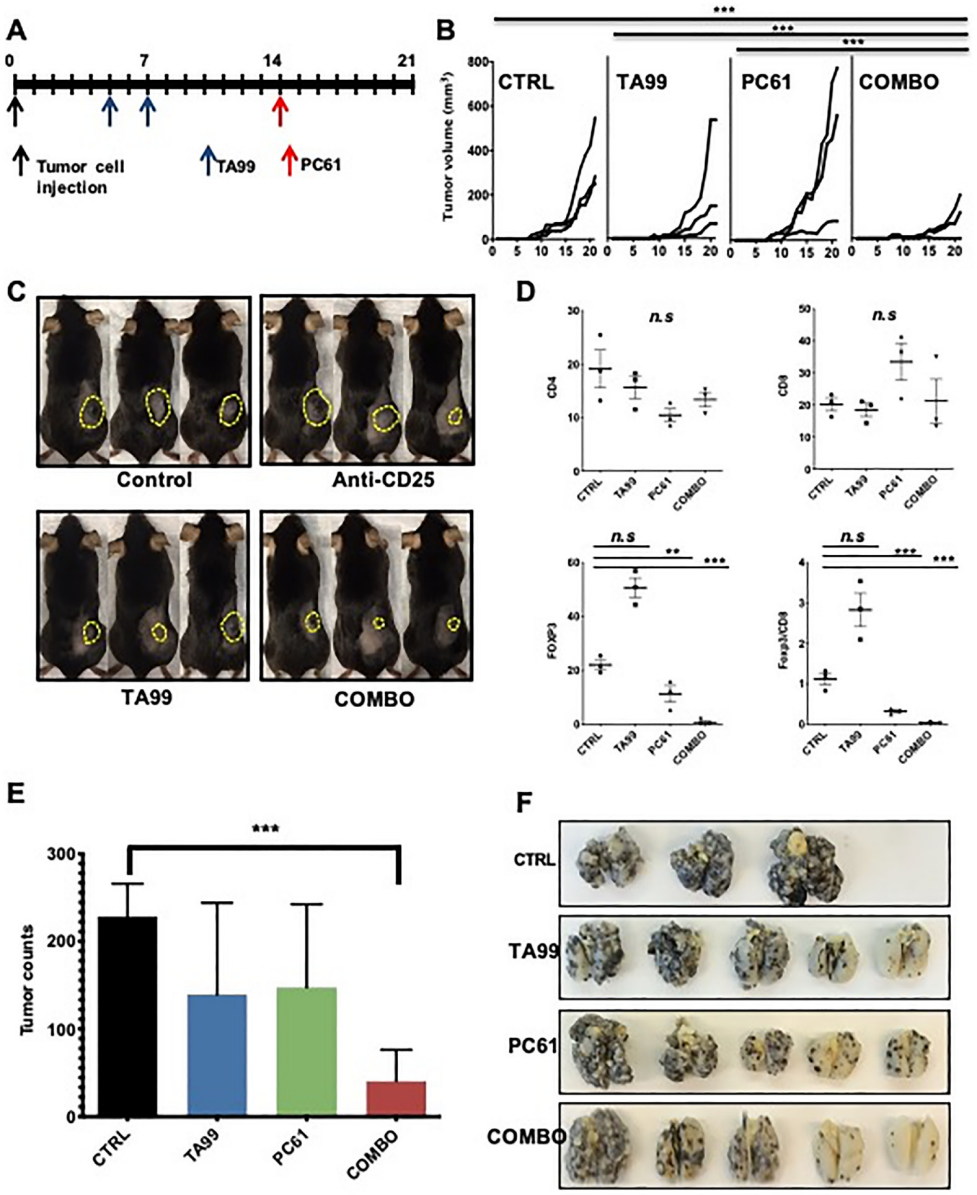
Depletion of Treg cells increased the anti-melanoma effects of TA99 mAb. C57BL/6 mice were treated with TA99 (anti-TYRP1) and PC61 (anti-CD25 depleting antibodies) beginning five days after *s.c.* or *i.v.* tumor cell injection (**A**). Subcutaneous tumor size was measured three times per week. Growth curves over time after grafting (days) are shown (**B**), and representative images of mice in different groups are shown (**C**). Flow cytometry analysis of these tumors showed a significant decrease in Treg cells infiltration (**D**). In the metastasis model, lungs were harvested on day 21 after tumor cell injection and metastatic nodules were counted (**E**). Images of lungs fixed in Fekete’s solution are shown (**F**). The combination of TA99 mAb with antibody-dependent Treg depletion (anti-CD25) reduced the growth of subcutaneous tumors, and lung metastases burden; mean ± SEM is shown. Significance was determined by two-way ANOVA with Bonferroni correction (B and D), or unpaired *t* test (E). *n* = 3 mice per group in (B–D), and 5 mice per group in (E) (representative from two experiments with similar results). ^**^ *p* < 0.001; ^***^ *p* < 0.0001.

The passive administration of anti-tumor antibodies activates adaptive tumor specific immunity in the host, which can be downregulated by tumor-regulated inhibitory processes such as the accumulation of Treg cells. Thus, to enhance the therapeutic effects of anti-tumor antibodies by removing immune suppressive regulatory signals, we first tested the combination of anti-tumor antibodies [TA99; anti- tyrosinase-related protein-1 (TYRP1) monoclonal antibodies] with antibody-mediated depletion of Treg cells in the B16 mouse model of melanoma. Seven-week-old female C57BL/6 mice were inoculated with 5 × 10^4^ B16 cells (B16) in the right flank (s.c.), and injected (i.p.) with TA99 monoclonal antibodies (mAb) on days 5 and 7 after tumor inoculation and a single dose of Treg depleting antibodies [anti-CD25 (PC61 mAb)] on day 14 (Figure 1A). Seven days after a single dose of PC61, we observed a significant depletion of CD25^+^ T cells in the spleen of treated mice (Supplementary Figure 1A). Administration of TA99 in combination with antibody-mediated depletion of Treg cells resulted in a significant reduction in subcutaneous B16 melanoma growth (*p* < 0.0001) (Figure 1B and 1C). Treatment with PC61 mAb, alone or in combination with TA99 antibodies, resulted in the expected reduction in Treg cells infiltrating tumors (Figure 1D). We did not observe a significant difference in CD4^+^ or CD8^+^ cells infiltrating these tumors; but there was a decreased Foxp3/CD8 ratio in tumors treated with PC61 (Figure 1D). These tumors did not show diminished proliferation as demonstrated by Ki67 immunostaining (Supplementary Figure 1B), suggesting increased tumor cell death. In contrast, depletion of CD25^+^ cells shortly after tumor injection (day 4) resulted in increased tumor growth with no synergistic effect with TA99 administered on days 5 and 7 as observed before (Supplementary Figure 1C and 1D).

We also tested the combination of TA99 with PC61 in the B16 mouse model of lung metastases. After intravenous inoculation of 2 × 10^5^ B16 tumor cells, we administered monotherapy and combination therapy with TA99 and PC61 according to the schedule shown in Figure 1A. Briefly, TA99 was administered (i.p.) on days 5 and 7 after tumor cell injection, and a single dose of PC61 was administered on day 14 post-tumor inoculation. On day 21 post-tumor cell inoculation, the lungs were harvested and fixed in Fekete’s solution and tumors counted blindly under a dissection scope. The combination of TA99/PC61 resulted in a significant reduction in the burden of pigmented nodules in the lungs (*p* = 0.002) (Figure 1E and 1F) without any significant changes in tumor burden in the monotherapy groups. Taken together, these results show that elimination of immunosuppressive signals from T regulatory cells after passive administration of specific anti-tumor antibodies improved the therapeutic outcome in the B16 melanoma models of solid tumors and lung metastases.

### Anti-4-1BB/CD137 agonistic mAb increases the anti-tumor effects of TA99 in B16 mouse melanoma

**Figure 2:**
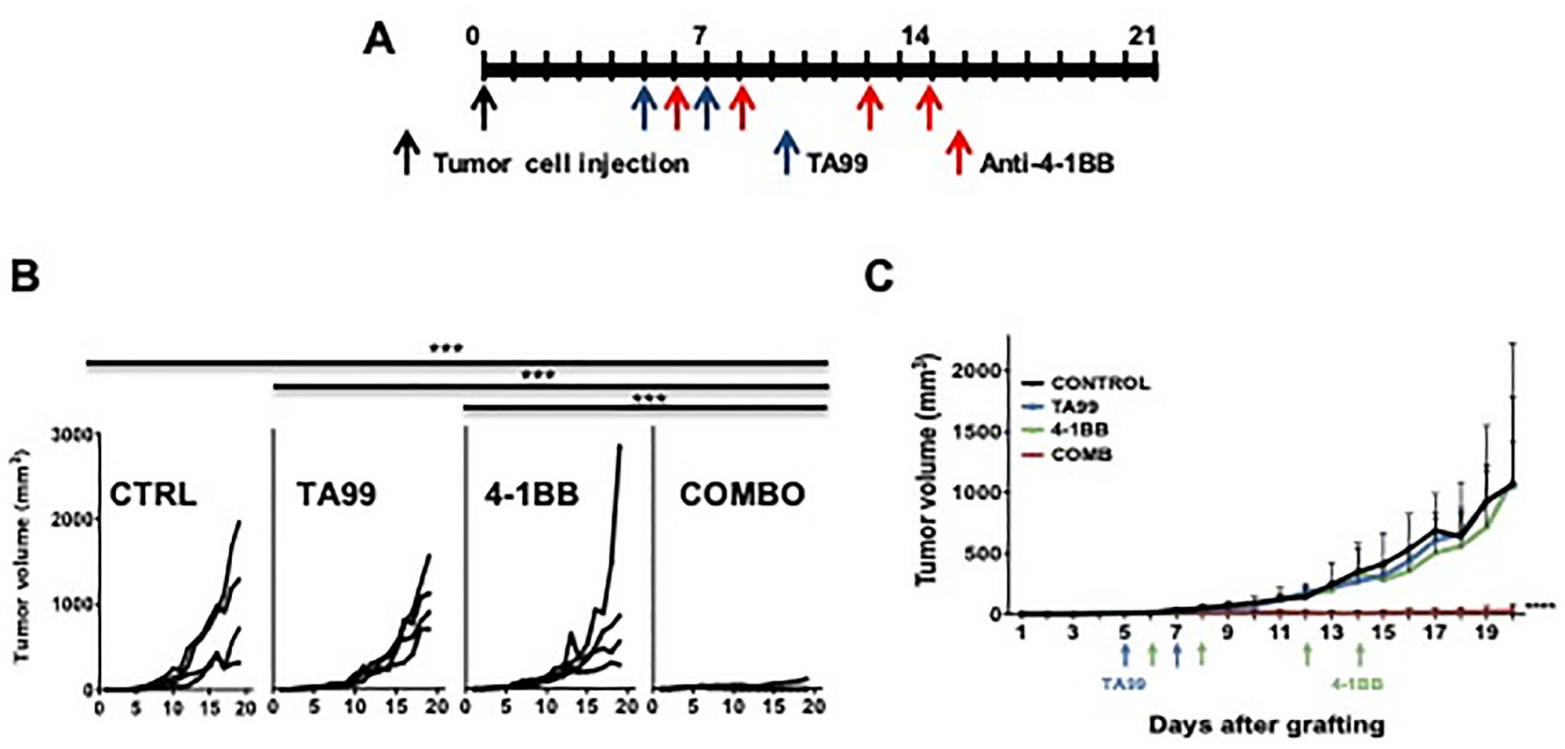
Anti-4-1BB/CD137 agonistic mAb enhanced the anti-tumor effects of TA99 in B16 subcutaneous melanomas. C57BL/6 mice were treated with TA99, anti-4-1BB agonistic antibodies or the combination beginning five days after B16 tumor cells subcutaneous inoculation (**A**). Combination treatment with TA99 and anti-4-1BB mAb led to eradication of B16 subcutaneous tumors (**B**). 5 × 10^4^ B16 cells were injected (*s.c.*) and tumors were allowed to reach 65–80 mm^3^, and then treated as in (A), and combination treatment resulted in a significant reduction in tumor growth (**C**). Data are presented as the mean ± SEM, and significance was determined by two-way ANOVA with Bonferroni correction (*n* = 4 mice per group in [B], and 5 mice per group in [C]). ^*^ *p* < 0.05; ^***^ *p* < 0.0001.

We observed that treatment of mice bearing subcutaneous melanomas with TA99 resulted in an increase in 4-1BB^+^ cells infiltrating the tumors (Supplementary Figure 2), suggesting that these tumors may be amenable to immunotherapy with anti-4-1BB agonist mAb, which may also enhance the anti-tumor effects of TA99 when administered in combination. Thus, we treated subcutaneous B16 mouse melanomas with TA99, anti-4-1BB agonist mAb, or the combination, starting on day 5 after tumor inoculation (Figure 2A), and followed tumor growth for up to 21 days. The combination therapy with TA99 and anti-4-1BB agonistic mAb resulted in eradication of subcutaneous B16 melanomas (Figure 2B). In contrast, no effect on tumor growth was observed when these mAbs were given as single-agent therapy. Furthermore, the combination of TA99 with anti-4-1BB mAb also resulted in a significant reduction in tumor size when treatment was started after the subcutaneous B16 tumors reached a volume between 65 and 80 mm^3^ (Figure 2C). Thus, eliminating regulatory events (via antibody-mediated Treg depletion; Figure 1) or inducing activation of immune effectors (using anti-4-1BB agonistic mAb; Figure 2), enhanced the therapeutic effects of the TA99 anti-tumor monoclonal antibody (anti-TYRP1) in the B16 mouse model of melanoma.

### Immune checkpoint blockade increases the efficacy of TA99 mAb in the B16 model of melanoma

**Figure 3:**
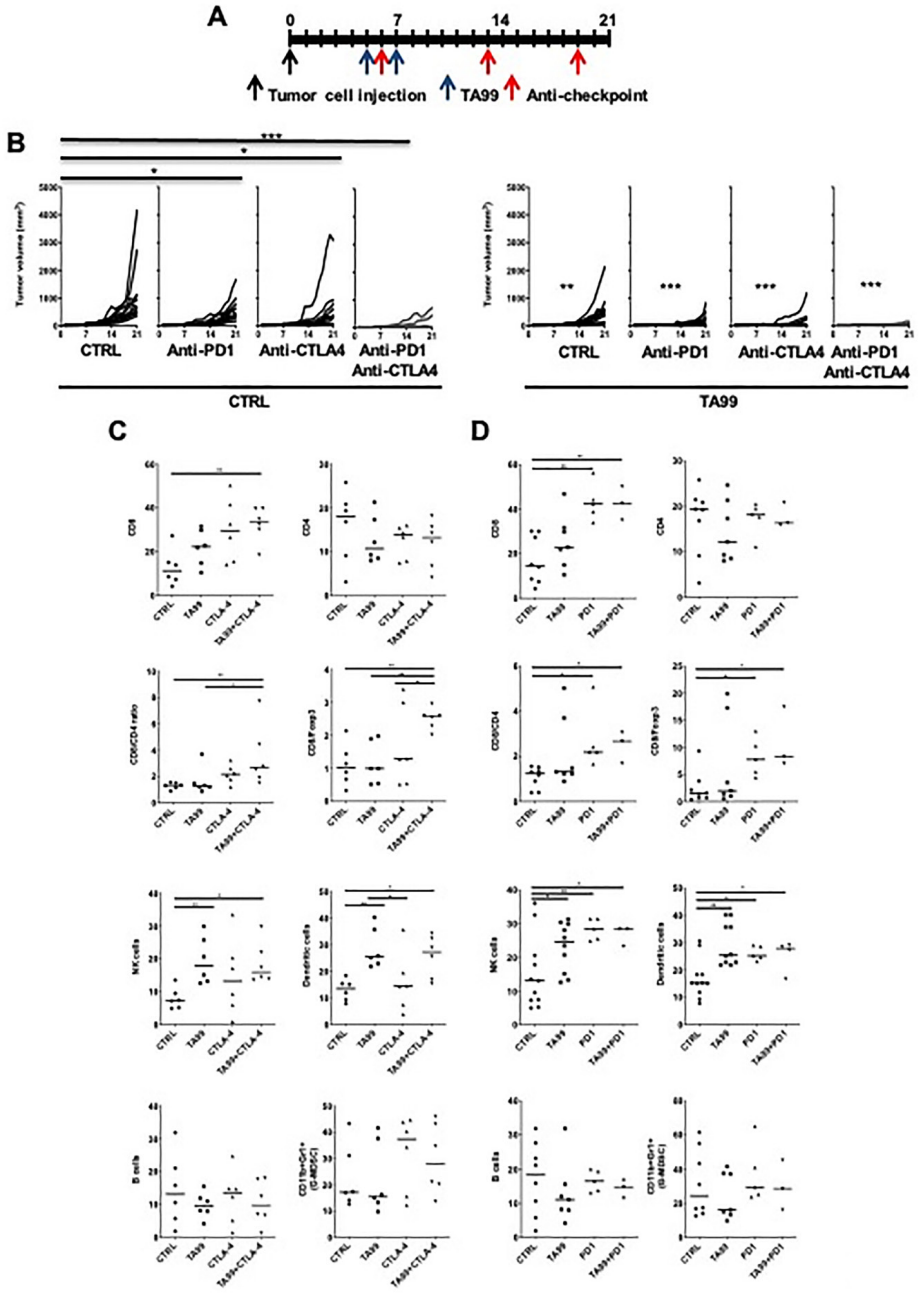
ICB enhanced the anti-tumor efficacy of TA99 in B16 subcutaneous melanoma. Treatment schedule of B16 melanoma bearing C57BL/6 mice with TA99 mAb alone or in combination with anti-PD1 or anti-CTLA4 (**A**). TA99 or ICB therapy alone resulted in modest tumor growth inhibition, which became highly significant when these therapeutic agents were combined (**B**). Differences in tumor growth were determined by two-way ANOVA with Bonferroni correction (*n* = 10 mice per group). ^*^ *p* < 0.05; ^**^ *p* < 0.001; ^***^ *p* < 0.0001. Left panel shows the comparison of different treatment with the untreated control group. Statistical significance on the right panel shows comparison with the no TA99 control group (left). At the end point, tumors treated with TA99 and anti-CTLA4 (**C**), and anti-PD1 (**D**) were collected after euthanasia and immunophenotyped by FACS analysis. Individual tumors are represented in the scatter plots and the median is shown. ^*^ *p* < 0.05; ^**^ *p* < 0.001; ^***^ *p* < 0.0001.

Therapy with immune checkpoint blockers such as ipilimumab (anti-CTLA4), and nivolumab and pembrolizumab (anti-PD1), has been established as the standard-of-care for melanoma with a significant improvement in OS. Blockade of CTLA4 has proven effective in melanoma and other tumors, and has a profound effect on the Treg population. We found that elimination of Treg cells improves the efficacy of TA99 in the B16 model of melanoma. Thus, to test the hypothesis that the elimination of regulatory signals by immune checkpoint blockade have an enhanced therapeutic effect with anti-tumor antibodies, we treated C57BL/6 mice bearing subcutaneous B16 tumors with TA99 mAb in combination with anti-immune checkpoint mAbs including anti-PD1 (Programmed Death 1) and/or anti-CTLA4 (Cytotoxic T Lymphocyte Associated antigen 4; IgG2b depleting antibody) as indicated (Figure 3A). Single-agent treatment with TA99 or either of the anti-immune checkpoint mAbs alone induced a modest reduction in subcutaneous melanoma tumor size (Figure 3B). However, when we tested combination treatment with TA99 and either anti-CTLA4 or anti-PD1, we observed a significant reduction in tumor growth compared to control animals or those that received monotherapy (Figure 3B). Furthermore, considering that current standard-of-care strategies in melanoma often include a combination of anti-PD1 and anti-CTLA4 therapy, we treated B16 tumor bearing mice with this double combination. We observed a significant reduction in volume when compared with the control or the single treatment groups (*p* < 0.0001). In order to investigate whether the administration of the combination of ICB further enhance the therapeutic effect of TA99, we tested the triple combination. Here, we observed the complete eradication of tumors (Figure 3B), demonstrating that the combined ICB therapy dramatically enhanced the therapeutic efficacy of TA99 antibodies in melanoma.

We next analyzed the composition of the immune infiltrates in tumors treated with TA99 in combination with anti-PD1 or anti-CTLA4 by flow cytometry (Figure 3C and 3D and Supplementary Figure 3). Tumors treated with TA99/anti-CTLA4 showed an increase in CD8^+^ cells and the CD8^+^/Foxp3^+^ cell ratio (Figure 3C). In addition, this combination resulted in a greater infiltration of NK1.1^+^ cells; however, a similar increase was reached when the single-agent therapy with TA99, anti-CTLA4 and anti-PD1was used alone (Figure 3C and 3D). Furthermore, treatment with TA99 alone and in combination with anti-CTLA4, but not anti-CTLA4 alone, induced an increase in the percent of dendritic cells (CD11b^+^CD11c^+^) infiltrating B16 subcutaneous melanomas (Figure 3C).

When we treated B16 tumors with anti-PD1 antibodies, we observed an increased CD8^+^ infiltrate that was also reflected as an increase in the CD8^+^/Foxp3^+^ cell ratio. We also found an increase in the dendritic cells infiltrating these tumors. However, no additional increase was found when the combination of TA99/anti-PD1 was tested (Figure 3D). We did not find differences in B cells (CD19^+^), CD4^+^ T cells or G-MDSC (CD11b^+^Gr-1^+^) (Figure 3C and 3D) in any of the experimental groups. These observations suggest that DC and their antigen presentation capabilities, as well as the potential activation of CD8^+^ T cells, may play a role in the increased anti-tumor effect of the therapeutic combinations tested.

**Figure 4:**
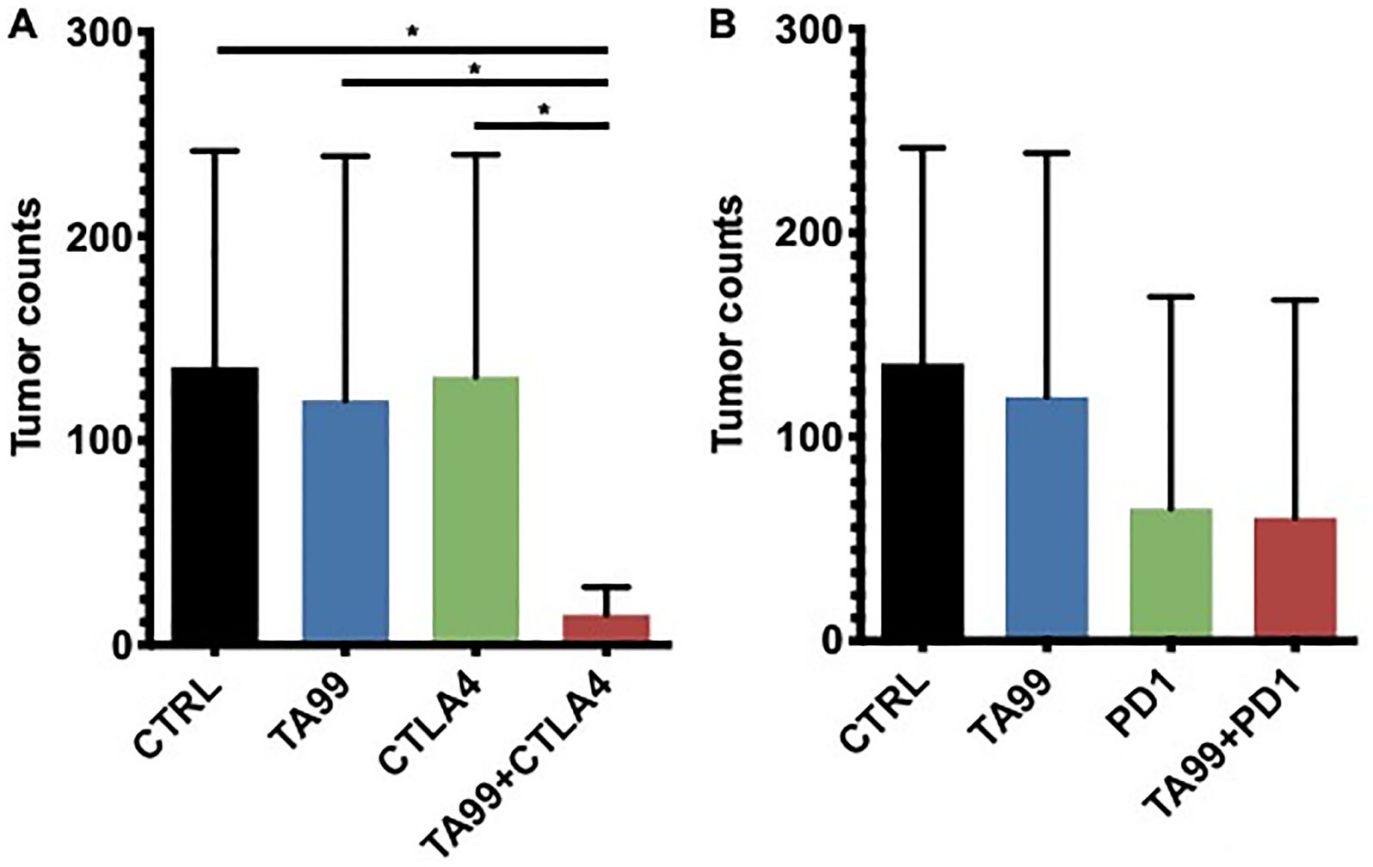
Combination therapy TA99/ICB reduced the lung tumor burden in the B16 model of metastases. C57BL/6 mice were inoculated B16 cells via tail vein injection, and treatment with anti-CTLA4 (**A**) or anti-PD1 (**B**) alone or in combination with TA99 was administered as described in Figure 3A. After 21 days, lungs were harvested and fixed, and metastatic nodules counted. The combination of TA99 mAb with immune checkpoint blockade reduced the lung metastases burden. Mean ± SEM is shown. ^*^ *p* < 0.05.

Additionally, we tested the combination of TA99 with ICB in the B16 model of lung metastases (Figure 4). Treatment with TA99, anti-CTLA4 (Figure 4A) or anti-PD1 (Figure 4B) mAbs as single agents did not show any significant difference in tumor burden between groups or when compared with untreated controls. However, the combination treatment with TA99 and blockade of CTLA4 resulted in a significant reduction in the number of melanomas in the lungs of the majority (4/5) of treated animals (Figure 4A). In addition, as expected, treatment with a combination of anti-CTLA4 and anti-PD1 mAb reduced the number of lung metastases in all the animals, however, we did not observe additional reduction in lung metastases as a result of a triple combination with TA99 (Supplementary Figure 4).

### MEK inhibition enhances the therapeutic efficacy of TA99 anti-tumor antibodies in B16 and BRAF mutant mouse melanomas

**Figure 5:**
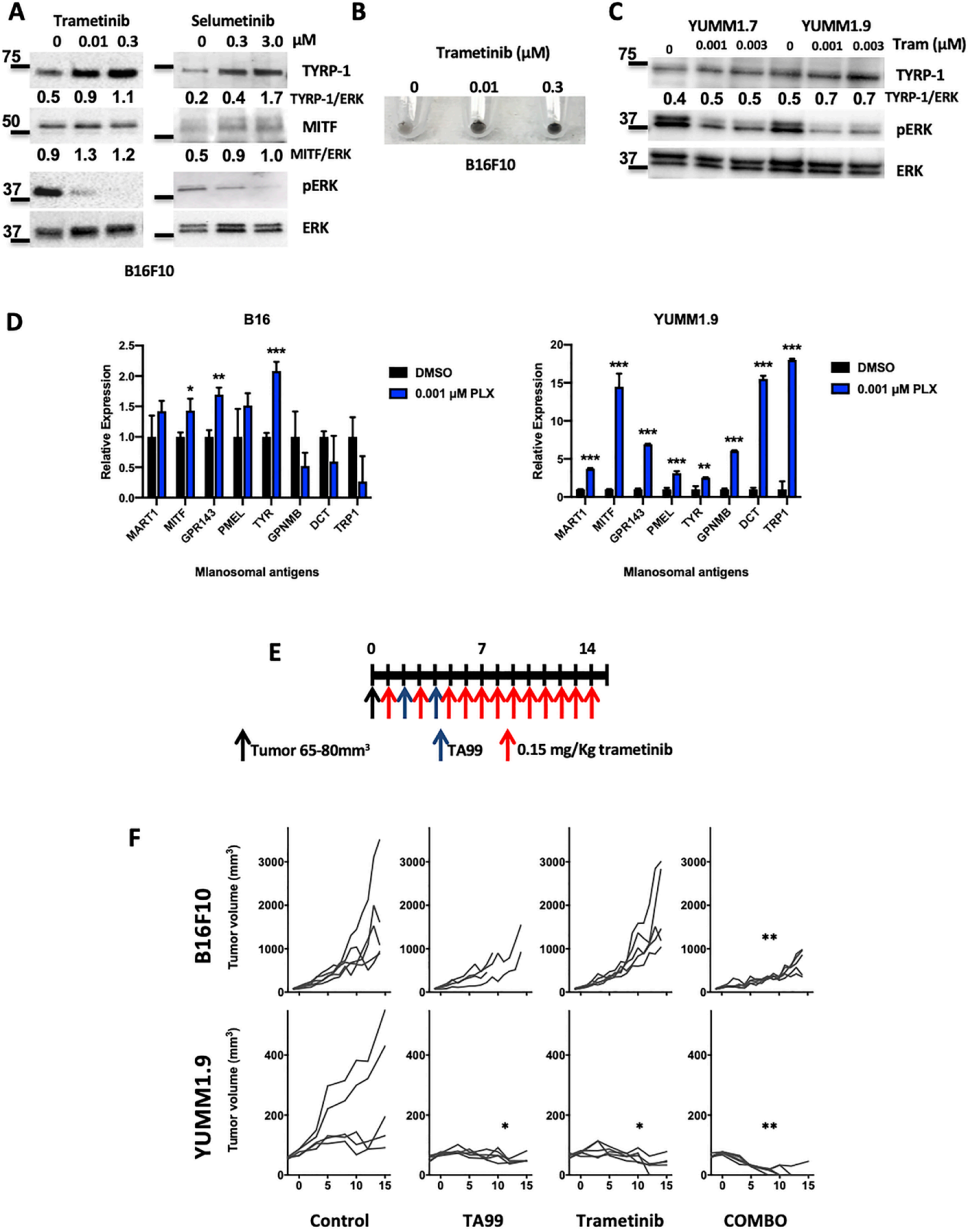
MEK inhibition enhances the anti-tumor effect with TA99 mAb. B16 cells were treated with MEKi for the indicated times and protein lysates were subjected to western blot analysis. MEK inhibition (as shown by dephosphorylation of ERK) resulted in increased levels of TYRP1 and MITF (**A**) and increased pigmentation (**B**). Trametinib also induced increased levels of TYRP1 in BRAF^V600E^-mutant YUMM cells (**C**). RNA was prepared after treatment with trametinib, and increased expression of different melanosomal antigens was observed by qRT-PCR analyses. Relative expression of melanosomal antigens from technical triplicates is shown. Grey bars represent DMSO-treated controls, and blue bars represent melanoma cells after treatment with trametinib. Melanosomal antigens measured are: MART, MITF, GPR143, PMEL, TYR, GPMNB, DCT and TYRP1 (**D**) ^*^ *p* < 0.05; ^**^ *p* < 0.001; ^***^ *p* < 0.0001. Combined treatment with trametinib and TA99 (**E**) resulted in an enhanced anti-melanoma effect in both B16 and YUMM1.9 subcutaneous tumors in C57BL/6 mice (**F**). Differences in tumor growth were determined by two-way ANOVA with Bonferroni correction (*n* = 5 mice per group). ^*^ *p* < 0.05; ^**^ *p* < 0.001. Comparison between the corresponding treatment group and the untreated control is shown.

It has been established that inhibition of the MAPK pathway with BRAF and MEK inhibitors induces de-differentiation of BRAF mutant human melanoma cells. This process is characterized by an increased expression of melanosomal antigens, including TYRP1, the target for TA99 antibodies. Thus, we next tested the *in vitro* effects of MEK inhibitors (MEKi) on B16 cell viability by MTS assays. Selumetinib (AZD6244) and trametinib partially reduced the viability of B16 in a dose dependent manner (Supplementary Figure 5A). We also found a dose-dependent increase in the levels of both TYRP1 and MITF (melanosomal antigens) upon treatment of B16 cells with either selumetinib (6 h) or trametinib (24 h), which correlated with an effective downregulation of the MAPK pathway as demonstrated by dephosphorylation of ERK (Figure 5A). Furthermore, treatment with the MEKi trametinib induced increased pigmentation in B16 mouse melanoma cells (Figure 5B and Supplementary Figure 5B). We also tested the trametinib-mediated induction of melanosomal antigens in the trametinib-sensitive BRAF mutant cell lines YUMM1.7 and YUMM1.9, and found increased expression of TYRP1 (Figure 5C). Moreover, using qPCR, we found increased expression of TYRP-1, MITF and other melanosomal antigens (Figure 5D).

Since MEK inhibition resulted in the increased expression of the TA99 mAb target itself (TYRP1), as well as other melanosomal antigens, we treated B16 and YUMM1.9 subcutaneous tumor bearing mice with a combination with trametinib and TA99 (Figure 5E) and followed tumor growth. As predicted, when we treated the relatively trametinib-resistant B16 melanomas with TA99/trametinib, tumor growth in the single-agent treatment groups showed the same pattern as the control group. However, treatment with the combination TA99 and trametinib resulted in a significant reduction in tumor growth (Figure 5F, top). Moreover, after treatment of the more trametinib sensitive YUMM1.9 tumor bearing animals, we observed a significant reduction in tumor growth with either of the single-agent treatments and the eradication of subcutaneous melanomas in the majority of the animals treated with the combination of TA99 and trametinib (Figure 5F, bottom).

**Figure 6:**
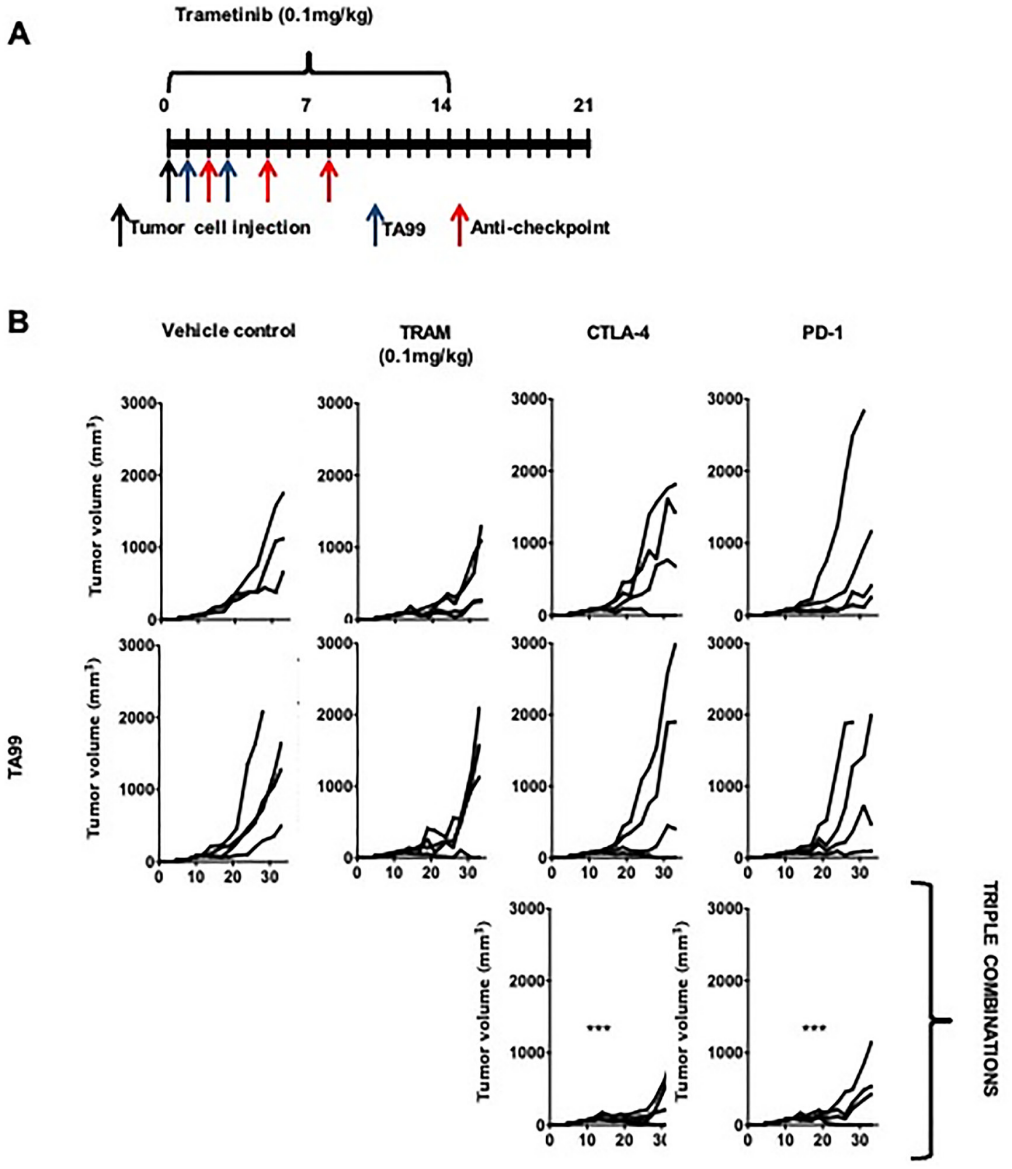
TA99 anti-tumor antibodies improved the outcome of treatment with combined targeted therapy and ICB in BRAF^V600E^-mutant subcutaneous YUMM melanoma. C57BL/6 mice bearing YUMM1.7 subcutaneous melanoma were treated with MEKi, anti-immune checkpoint antibodies, or TA99 as single-agent therapies or in different simultaneous combinations (**A**) and differences in tumor growth were determined. The triple combination showed a significantly increased latency and reduced tumor size (**B**). Differences in tumor growth were determined by two-way ANOVA with Bonferroni correction (*n* = 4 mice per group). ^***^ *p* < 0.0001. Both triple combinations show a significant increased control of tumor growth when compared with untreated control and with single and double agent treatment.

Based in our observations that the therapeutic effects of the anti-tumor antibodies TA99 can be enhanced by both ICB (Figures 3 and 4) and MEKi targeted therapy (Figure 5), we tested the triple combination in the BRAF mutant YUMM1.7 cell line (Figure 6A). This combination treatment with TA99, trametinib and ICB (with anti-PD1 or anti-CTLA4) mAb resulted in a significant reduction of tumor size compared to the control groups (Figure 6B).

## DISCUSSION

Targeting tumor antigens with specific antibodies, such as anti-CD20 (rituximab), anti-HER2 (trastuzumab), anti-EGFR (cetuximab), is well-established and has been relatively successful in a number of cancers, via interference with cellular signaling, complement-mediated lysis, antibody-dependent cell-mediated cytotoxicity (ADCC), and/or antibody-dependent cell phagocytosis (ADDP) [12–14, 16, 17]. However, the use of these unmodified antibodies during the therapy of advanced solid tumors such as breast cancer, metastatic colorectal cancer and head and neck squamous cell carcinomas as monotherapy, result in a high proportion of tumors with primary and acquired resistance, and relatively low rates of lasting therapeutic responses [18–20].

Here, using mouse models of melanoma, we investigated the use of the TA99 mAb directed against TYRP1, a surface protein not involved in cellular signaling, whose administration we and others showed previously induced long-lasting FcR-dependent tumor specific immunity in the host, with kinetics consistent with an induced adaptive immune response against the tumor [12, 13, 21]. It has been shown that the use of TA99 enhances the beneficial effects of TYRP1 DNA [22] and peptide vaccination [23]. Further, TA99 in combination with IL-2 and T cell vaccines is effective for the elimination of established tumors in the B16 model of melanoma [24].

Successful activation of the adaptive immune response by TA99 is also strictly regulated *in vivo* by inhibitory processes mediated by Treg cells and immune checkpoints. Here, we demonstrated that elimination of Treg strongly enhanced the anti-tumor effects of TA99 in the B16 models of subcutaneous solid tumors and lung metastases, suggesting the potential therapeutic advantage of the combination of specific anti-tumor antibodies with therapeutic strategies aimed at inhibiting other immunosuppressive processes, such as immune checkpoint blockade. Nonetheless, our (Left). observations indicated that elimination of CD25^+^ cells at early time points, i.e., day 4 after tumor cell inoculation induced a more rapid and larger outgrowth of B16 subcutaneous tumors. In agreement, it has been previously shown that CD4^+^ T cell help and IL-2 signaling were linked via CD25 up-regulation for the control of the expansion and differentiation of antigen-specific effector CD8^+^ T cells [25]. Thus, we postulate that the early elimination of CD25^+^ T cells in our model results in the impairment of effector anti-tumor responses. Together, our findings indicate that the combination therapy with TA99 and the elimination of the regulatory signals resulting from the activation of anti-tumor immune responses could be an effective therapeutic strategy for the management of malignant melanoma.

Previous studies showed that the combination of TA99 with immunostimulatory molecules, such as toll-like receptor ligands and IL-2 has therapeutic effect dependent on a coordinated effect of the innate and adaptive anti-tumor immune response involving CD8^+^ T cells, NK and macrophages, and the expression of activating FcγR, which increases the success rates of tumor-targeting TA99 in eliminating tumor cells [24, 26, 27]. Interestingly, we found that treatment with TA99 results in an increase in 4-1BB^+^ (CD137) cells, thus we postulated that the activation of the adaptive immune response induced by the treatment with TA99 could be enhanced by further cell activation to drive a robust effector response instead of tolerance. In line with previous reports in which the therapeutic activity of TA99 against established B16 tumors was enhanced with antibody-cytokine fusion (TA99-TNF) [28], we showed that agonist antibodies to 4-1BB, a member of the TNFR family expressed on activated T and NK cells, synergized the anti-tumor effects of TA99. Further, it has been reported that agonistic activation of 4-1BB induced CD4^+^ and CD8^+^ T cell activation, and prevented their activation-induced death, making these agonist antibodies an ideal therapeutic candidate to enhance anti-tumor immunity [29, 30]. In fact, preclinical and clinical studies have shown that the combination of the anti-EGFR antibody cetuximab with anti-4-1BB agonist leads to tumor resolution and prolonged survival, likely dependent on enhanced NK cell degranulation and cytotoxicity, in head and neck and colorectal cancers [29]. Together, we showed activation of the anti-tumor immune response with anti-4-1BB agonist antibodies can enhance the therapeutic effects of unmodified anti-tumor antibodies directed against non-signaling molecules such as TA99.

The successful activation of innate and adaptive immune responses upon therapy with anti-tumor antibodies may be hindered by other regulatory mechanisms such as immune checkpoints. Monoclonal antibodies directed at blockade of immune checkpoint inhibitors are already in clinical use and have had a profound impact in the treatment of metastatic melanoma and other cancers. Here, we showed that the combination of ICB with anti-PD1 or anti-CTLA4 antibodies with specific anti-tumor antibodies (TA99) results in an increased efficacy against B16 subcutaneous and lung melanomas, in agreement with previous reports showing that therapy with anti-HER2 antibodies synergizes with immune checkpoint blockade with anti-PD1 in mouse models of breast cancer [31]. Furthermore, as predicted from the current clinical data on anti-CTLA4 and anti-PD1 therapeutics [32, 33], we found that this combination resulted in a significant reduction of B16 tumors, which when combined with TA99 mAb therapy in a triple combination, resulted in eradication of solid subcutaneous tumors. It has been reported that eradication of large B16 tumors requires TA99, anti-PD1, and IL2 and a potent Tcell vaccine [24], however those tumors may be extremely aggressive since they are the result of a very high number of seeding B16 cells (1 × 10^6^ vs. 5 × 10^4^ in our study). Moreover, in the B16 lung metastasis model, when we administered double ICB, we observed a significant reduction in the number of tumoral masses, however, the triple combination treatment did not improve the anti-tumor effect, as predicted from our observations in the solid tumors. This highlights the need for further studies to determine the correct timing and sequence of different therapeutic strategies, as suggested elsewhere [34].

Although we found that single treatment with TA99 did not result in significant changes in the recruitment of CD4^+^ and CD8^+^ T cells, the CD8^+^/Foxp3^+^ ratio was increased in the tumors treated with the combination TA99/ICB. In agreement, it has been described that high doses of TA99 at early time points delays tumor growth, which is associated with increase in intratumoral CD4^+^ and CD8^+^ effectors, but it does not prevent exhaustion [21]. Moreover, we showed that treatment with TA99 induced a greater DC infiltrate in B16 tumors. This finding is consistent with reports indicating that treatment with the anti-EGFR antibody cetuximab results in an increased NK cell mediated ADCC, which makes the tumor cells or their antigens more susceptible to phagocytosis by DC [16, 17]. Further, it has been reported that resistance to immunotherapy may be due in part to defective recruitment of DC, downregulation of antigen processing and presentation, and thus reduced cross-priming [24, 35–37]. In fact, the combination PD1/PDL1 blockade with agonist anti-CD27 in the B16 model resulted in an increased CD8+ T cell expansion and effector function. Additionally, varlilumab (anti-CD27 agonist) has shown the same in a humanized model of lymphoma [36]. Together, these findings underscore the potential for combination therapy with anti-tumor antibodies and ICB to improve therapeutic outcomes in patients with advanced melanoma.

There is evidence that chemotherapy and targeted therapy not only have direct cytostatic or cytotoxic effects on cancer cells, but also induce the activation of tumor-targeting immune responses, perhaps by increasing immunogenicity of malignant cells. Thus, the immunological effects of standard-of-care therapies can be desirable and useful during the establishment of successful combinatorial regimens [38]. It was previously described that BRAF and MEK inhibition induce the upregulation of a group of melanosomal proteins, known as the “melanosomal signature” in human BRAF^V600E^ cell lines and tumors [39–42], while activation of MAPK signaling reduces melanogenesis in B16 cells [43]. Accordingly, we found that *in vitro* treatment of the mouse BRAF^V600E^ cell lines YUMM1.7 and YUMM1.9, with the MEKi trametinib induced expression of the target of TA99 antibodies (TYRP1) and other melanosomal antigens. Moreover, B16 cells, which are relatively resistant to MEK inhibitors, also showed this response. We showed that MEKi-mediated induction of melanosomal antigens, particularly TYRP1, combined with the administration of anti-tumor antibodies resulted in an enhanced therapeutic effect in both the BRAF-WT and BRAF^V600E^ models. We postulate that this profound effect in the therapeutic outcome is due in part to MEKi-induced increase in tumor immunogenicity. The same phenomenon was previously described as a marker for positive response to anti-PD1 immunotherapy [44]. In addition, it was described that BRAF and MEK inhibition are capable of inducing a de-differentiation/re-pigmentation process depending on levels of MITF expression, especially in resistant cells [45]. Here, we demonstrated that increasing the expression of melanoma derived antigens with MEK inhibitors, in combination with an enhanced specific immune response through the use of anti-tumor antibodies, improved the response rates to ICB in the B16 and YUMM mouse models of melanoma. Thus, we postulate that inhibitors of the MAPK signaling pathway will reduce proliferation in sensitive cells and induce re-pigmentation in the survivors with an increased expression of TYRP1, which is the target of TA99 mAb, therefore contributing to their elimination in our model.

Our therapeutic strategy is highly relevant to the treatment of melanoma patients, given the specificity of the antigen and its conservation across species [28]. Notably, a fully human anti-TYRP1 monoclonal antibody (20D7) that induced strong ADCC and suppressed human and mouse melanoma growth in subcutaneous and metastatic models in immunocompromised mice was described in the literature [46]. Moreover, 20D7 mAb was tested in patients with advanced melanoma who progressed on at least one line of treatment (Flanvotumab, ImClone Systems). This phase I/Ib clinical trial showed that 20D7 was well tolerated (NCT01137006). In addition, one patient (1/27) experienced a complete response, 10 patients (47%) showed stable disease and 12 subjects (44%) had progressive disease [47]. In melanoma, the use of BRAF/MEK inhibitors, depending on the driver mutations of the tumors [48], showed an OS of over 9 months in BRAF mutant tumors [49]. Moreover, therapy with immune checkpoint blockade, i.e., ipilimumab (anti-CTLA4), nivolumab and pembrolizumab (anti-PD1), has been established as the standard-of-care for malignant metastatic melanoma. Immune checkpoint blockade has improved the median OS to 16.9 months, with a 4-year OS of 32.4% [15]. We postulate that using already approved forms of therapy (ICB and targeted therapy) may improve the efficacy of 20D7 unmodified anti-tumor antibodies.

Clinical trials with two different anti-4-1BB agonist antibodies, urelumab and utolimumab are ongoing [50]. Urelumab has shown high inflammatory liver toxicity, while utomilumab as a single agent has a more favorable safety profile [50, 51]. However, utomilumab has an overall objective response of only 3.8% in solid tumors, which underscores the need of combination strategies to capitalize on the therapeutic potential of these antibodies. Moreover, there is an ongoing phase 1B dose escalation clinical trial (NCT03364348) of anti-4-1BB agonistic in combination with trastuzumab (anti-HER2) in patients with HER2^+^ breast cancer.

Together with our preclinical data, these results invite further clinical investigation of unmodified anti-tumor antibodies in combination with ICB and targeted therapies, and may represent promising and innovative therapeutic interventions for the successful management of patients with advanced melanoma and other cancers.

## MATERIALS AND METHODS

### Mice

All animal experiments were conducted using 7-week-old female C57BL/6J mice purchased from The Jackson Laboratory. We used female animals to avoid scratching and wound healing as confounding variables in our studies. All animals were housed in groups of five animals/cage under a controlled environment of temperature and humidity and a 12 h light/dark cycle. Experimental procedures were carried out according to Columbia University institute of comparative medicine policies and an IACUC approved protocol.

### Cell lines and culture

B16F10 (B16) cells were obtained from the Columbia University Skin Disease Resource-Based Center (epiCURE), and cultured at 37°C in 5% CO_2_/95% air in DMEM supplemented with 10% FBS and 1% Penicillin/Streptomycin. YUMM 1.7 and YUMM 1.9 BRAF^V600E^ mutant mouse melanoma cells [52] were obtained from Dr. Marcus Bosenberg (Yale University), and maintained at 37°C in 5% CO_2_/95% air in DMEM/F12 medium supplemented with 10% FBS, 1% non-essential amino acids and 1% Penicillin/Streptomycin. Cell lines tested negative for Mycoplasma infection (PCR) at the beginning of the study.

### *In vivo* antibodies and inhibitors

The MEK inhibitor trametinib (GSK1120212) was purchased from Chemietek (Indianapolis, USA) and was dissolved to stock solutions in DMSO. For oral administration, a 1:10 dilution was prepared in hydroxypropyl methylcellulose and a maximum volume of 5 mL/kg of body weight was given daily. Monoclonal antibodies used for *in vivo* treatment were purchased from BioXCell, and prepared in sterile DPBS (Gibco) for injection. Animals received i.p. injections of 200 μg of anti-TYRP1 (TA99); 250 μg of anti-CD25 (PC61); 200 μg of anti-4-1BB/CD137 (3H3); 100 μg of anti-CTLA4 (9D9, IgG2b, depleting antibody), 200 μg of anti-PD1 (RMP1.14), or isotype control antibodies as indicated for each experiment.

### Tumor studies

The B16 model of melanoma has been described elsewhere [53]. Subcutaneous melanomas were generated by *s.c.* injection of 5 × 10^4^ B16 tumor cells or 1 x 10^5^ YUMM cells, and treatment was conducted as indicated in each experiment. Tumor growth was followed by measuring with a caliper, and volumes were calculated by using the formula (*d*^2^ × *D*) × 0.52, where *D* represents the greatest diameter and *d* represents the smallest diameter. Mice were euthanized when tumors exceeded 20 mm in diameter. For the lung metastasis model, 2 x 10^5^ B16 melanoma cells were injected in the tail vein and tumors were allowed to grow for 21 days with or without treatment. At the end point, animals were euthanized and the lungs were fixed in Fekete’s solution (55% ethanol; 3% formaldehyde; 4% acetic acid) for contrast. Melanoma nodules were counted under stereotactic microscope (when tumors were too numerous to be counted, > 250 was recorded). In all experiments, animal cages were randomly assigned to treatment groups (Excel random number generator).

### Flow cytometry analysis

Subcutaneous tumors were resected at the end point and single-cell suspensions were prepared and enriched in Percoll gradient. Cells were stained with monoclonal antibodies to CD45-brilliant violet™605, CD4-brilliant violet™510 (GK1.5), CD8a-brilliant violet™711, Ly-6G/Ly-6C(Gr-1)-brilliant violet™421, CD11c-PE/Dazzle™, I-A/I-E(MHCII)-PerCP/Cy5.5 (BioLegend), CD19-FITC, NK1.1-PE, Foxp3-APC, CD11b-Alexa Fluor^®^700, and F4/80-Pe/Cy7 (Thermo Fisher Scientific). Cells were acquired using a BD Biosciences Fortessa flow cytometer (Cancer Center Flow Core Facility and Columbia Center for Translational Immunology). Analyses were done using FCS Express 6 software.

### Western blot analysis

Cells were harvested in lysis buffer containing 50 mM Tris (pH7.4), 150 mM NaCl, 1 mM EDTA, 1% IGEPAL, 10 mM betaglycerophosphate, 50 mM sodium fluoride, 1 mM sodium orthovanadate, 1 ng/μL leupeptin, 1 ng/μL aprotinin, 1 ng/μL pepstatin A, 1 ng/μL AEBSF, and 10 nM calyculin A (Sigma). Twenty μg of protein were resolved by SDS-PAGE, transferred to PVDF membranes, and immunoblotted with antibodies to TYRP1 (BioXcell laboratories), MITF (Invitrogen), ERK, and p-ERK (Cell Signaling technologies).

### Quantitative RT-PCR

Mouse melanoma cells were treated with trametinib at the indicated concentrations. RNA from treated cells was isolated using RNeasy kit (Qiagen). cDNA was prepared using high-capacity cDNA reverse transcription kit (Applied Biosystems). Quantitative PCR was performed using PowerSYBR Green PCR Master Mix (Applied Biosystems) on the CFX96 Real-Time system (Bio-rad). Specific primer pairs for each gene were: TYRP1 (Fwd: CTTGGAGGTCCGTGTATTTG Rev: GACCGCATCAGTGAAAGTGT), MITF (Fwd: GCAAGAGGGAGTCATGCAGT Rev: GGGTCTGCACCTTAAGGACT), GPNMB (Fwd: GGGCATACATTCCCATCTCG Rev: AGTGTTGTCCCCAAAGTTCCA), MART1 (Fwd: CTTGATGGACAAAAGGCGTC Rev: AGCATTCTAAAGCGAAACACCG), GPR143 (Fwd: GGCTGCCTGGGAATCGTTAT Rev: AGC CCCCATCAGTCTCTCAT), DCT (Fwd: GTCCTCCACTCTTTTACAGACG Rev: ATTCGGTTGTGACCAATGGGT), PMEL (Fwd: TGACGGTGGACCCTGCCCAT Rev: AGCTTTGCGTGGCCCGTAGC), TYR (Fwd: ACTTACTCAGCCCAGCATCC Rev: AGTGGTCCCTCAGGTGTTCC). The specificity of the primers was confirmed by melting curve analysis. Results were normalized to GAPDH and fold change of each gene was calculated by 2^(ΔΔCt)^. Experiments were performed in triplicate.

### Cell viability assays

Changes in B16 cell viability upon treatment with MEK inhibitors *in vitro* were determined with the use of 3 - (4, 5 – dimethylthiazol – 2 - yl) – 5 - (3 - carboxymethoxyphenyl) – 2 - (4 - sulfophenyl) - 2H - tetrazolium (MTS) viability assays (Cell titer 96^Ò^ AQ_ueous_ Non-Radioactive Cell Proliferation Assay, Promega), following the manufacturer’s instructions. Briefly, 1 × 10^3^ B16 mouse melanoma cells per well were seeded in 96-well plates. After allowing attachment (overnight incubation), cells were treated with fresh media containing the indicated concentrations of the corresponding drugs or vehicle control. After 72 h incubation, 20 μL of MTS reagent was added and cells were incubated for additional 3 h before measuring absorbance at 490 nm.

### Quantification and statistical analysis

Data are presented as mean ± SEM. Statistical differences were determined using Student’s *t* test, Kruskal-Wallis, with Bonferroni’s post-test for multiple comparisons where appropriate. Statistical analyses were conducted using GraphPad Prism 6 software. Results were considered significant at *P* ≤ 0.05.

## SUPPLEMENTARY MATERIALS



## Abbreviations

ADCC: Antibody-dependent cellular cytotoxicity
APC: Antigen presenting cells
4-1BB: Tumor necrosis factor receptor superfamily member 9
BRAF: v-Raf murine sarcoma viral oncogene homolog B
CTLA4: Cytotoxic T-lymphocyte-associated protein 4
ERK: Extracellular signal-regulated kinases
ICB: Immune checkpoint blockade
mAb: Monoclonal antibody
MAPK: Mitogen-activated protein kinase
MEK: Mitogen-activated protein kinase kinase
MEKi: Mitogen-activated protein kinase kinase inhibitor
MITF: Microphthalmia-associated transcription factor
MTS: 3-(4,5-dimethylthiazol-2-yl)-5-(3-carboxymethoxyphenyl)-2-(4-sulfophenyl)-2H-tetrazolium
PD1: Programmed cell death protein 1
Treg: T regulatory cells
TYRP1: Tyrosinase-related protein 1
