# Supplementary material for: Improved therapeutic efficacy of unmodified anti-tumor antibodies by immune checkpoint blockade and kinase targeted therapy in mouse models of melanoma
Source: Oncotarget. 2021 Jan 19;12(2):66–80. doi: 10.18632/oncotarget.27868 (PMC7825641; doi:10.18632/oncotarget.27868)
Supplement: Supplementary file 1 [file oncotarget-12-66-s001.pdf]

Improved therapeutic efficacy of unmodified anti-tumor antibodies by immune checkpoint blockade and kinase targeted therapy in mouse models of melanoma

SUPPLEMENTARY MATERIALS

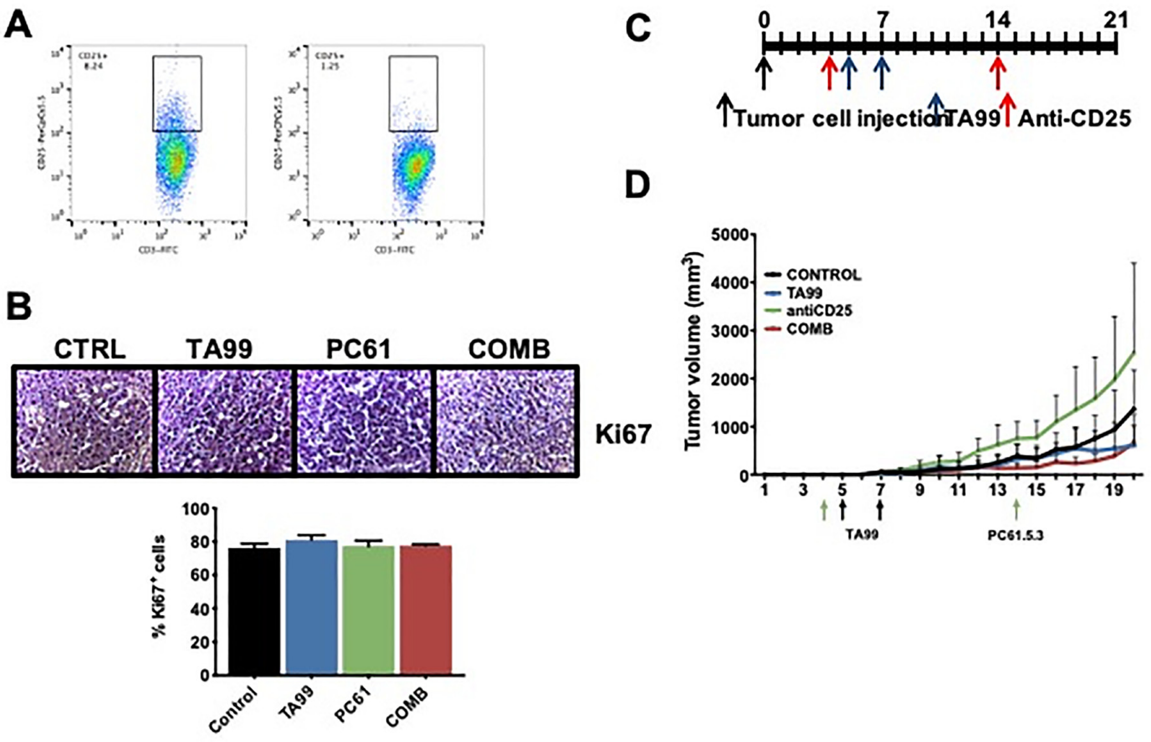

Supplementary Figure 1: Early depletion of CD25$^+$ cells increased tumor growth and negates the beneficial anti-melanoma effect in combination with TA99 mAb. Treatment of C57BL/6 mice with PC61 mAb (anti-CD25 depleting mAb) results in depletion of CD25$^+$ cells in the spleen of treated mice (**A**). Ki67 immunostaining of tumors (brown staining indicates Ki67 positive cells) from animals treated with TA99, PC61, or their combination, shows no difference in the proliferation rate in these tumors, suggesting that increased tumor cell death is responsible for the reduced tumor growth upon combination treatment with TA99 and PC61 mAb (B, top). Bottom panel in (**B**) shows quantitation of the percent of positive cells in three 20× random fields (*N* = 3 tumors/group) (B, bottom). In addition, we treated tumor (B16) bearing mice with PC61 one day before starting treatment with TA99, and at day 14 after tumor implantation (**C**). Here, we found that tumor growth in TA99-treated mice did not show a difference when compared with the combination group (**D**). Further, early depletion of CD25$^+$ with PC61 as a single agent in this setting resulted in B16 tumors growing faster and larger than any other experimental group; mean ± SEM is shown. Significance was determined by two-way ANOVA with Bonferroni correction. *n* = 4 mice per group.

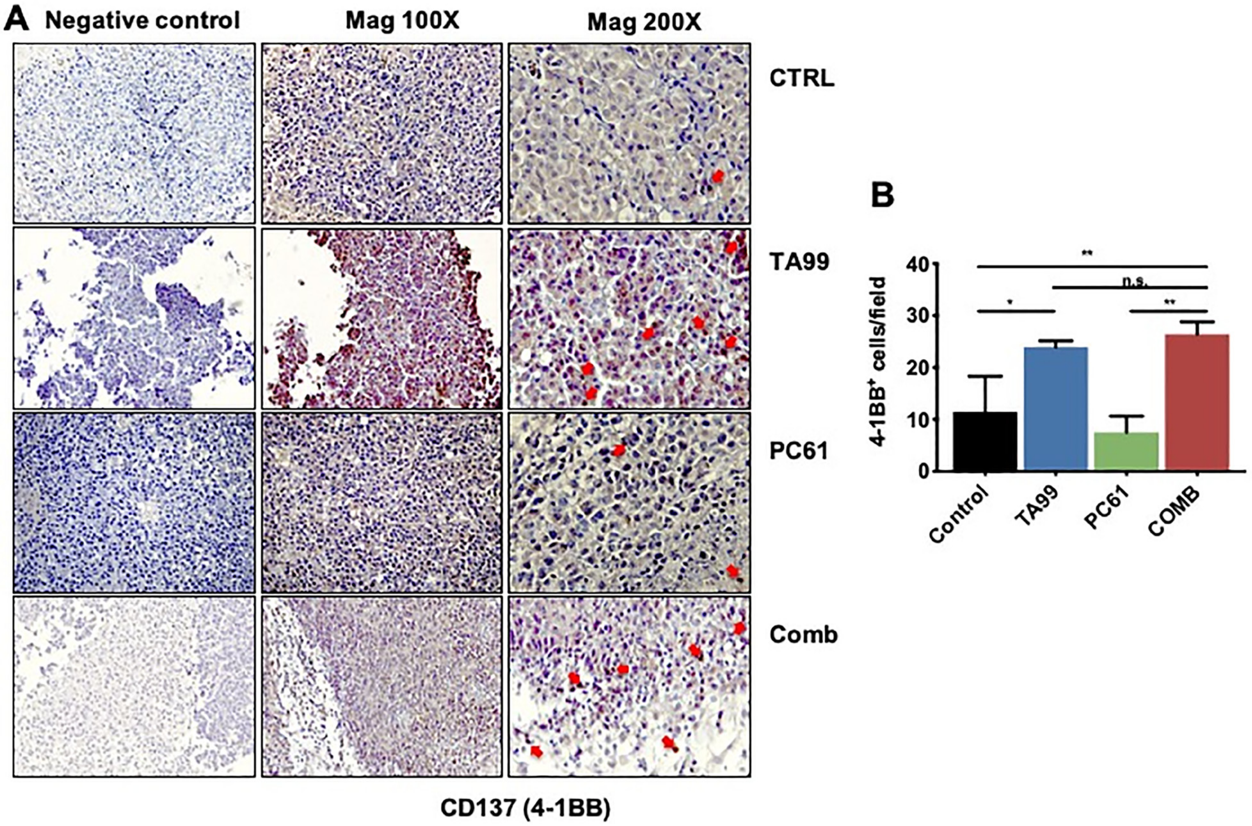

Supplementary Figure 2: Treatment with TA99 specific anti-tumor antibodies resulted in greater infiltration of 4-1BB/ $CD137^+$ cells. After treatment of tumor bearing C57BL/6 mice with TA99, PC61,8 or the combination of both, B16 subcutaneous tumors were harvested, fixed in formalin, embedded in paraffin, and sectioned. Five-µm sections were immunostained for 4-1BB (**A**). The increase in $4\text{-}1BB^+$ cells (brown staining indicates 4-1BB positive cells; arrows point to some positive cells) infiltrating tumors from animals treated with TA99 led to treatment with anti-4-1BB agonistic mAb to improve the therapeutic outcome. Two different magnifications (100 and 200×) of representative images are shown. 4-1BB positive cells per 10× field were counted in 3 random fields per section (**B**). n.s. not significant; $^*p < 0.05$; $^{**}p < 0.001$.

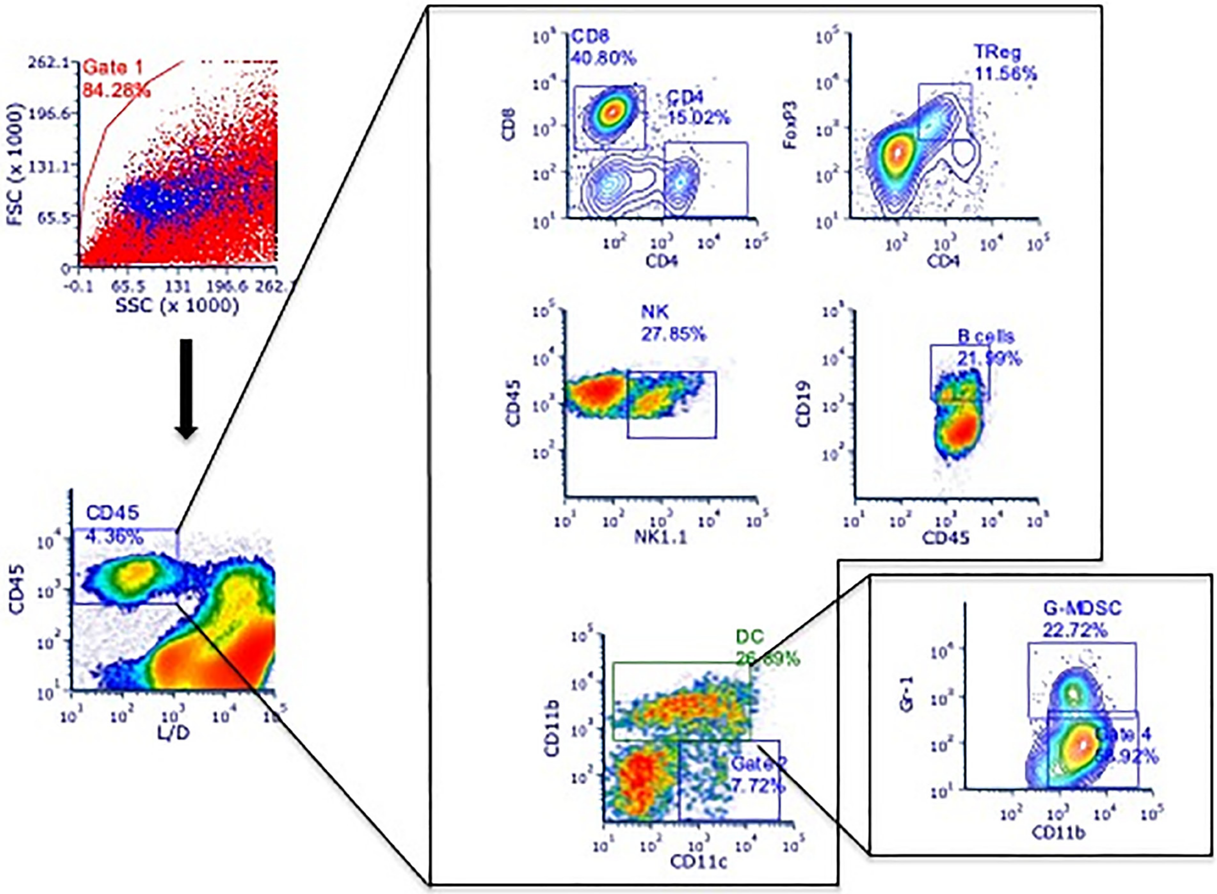

Supplementary Figure 3: Flow cytometry analysis strategy. B16F10 cells were grown as subcutaneous tumors in C57BL/6 mice and tumors were harvested and single cell suspensions were submitted to percoll gradient separation. Cells were then stained and acquired in a BD Fortessa instrument. $CD45^+$ cells were gated and analyzed for CD4/CD8, CD4/Foxp3 (Treg cells), NK1.1 (NK cells), CD19 (B cells), and CD11b/CD11c. The $CD11b^+CD11c^+$ gate was analyzed for expression of Gr-1 (G-MDSC).

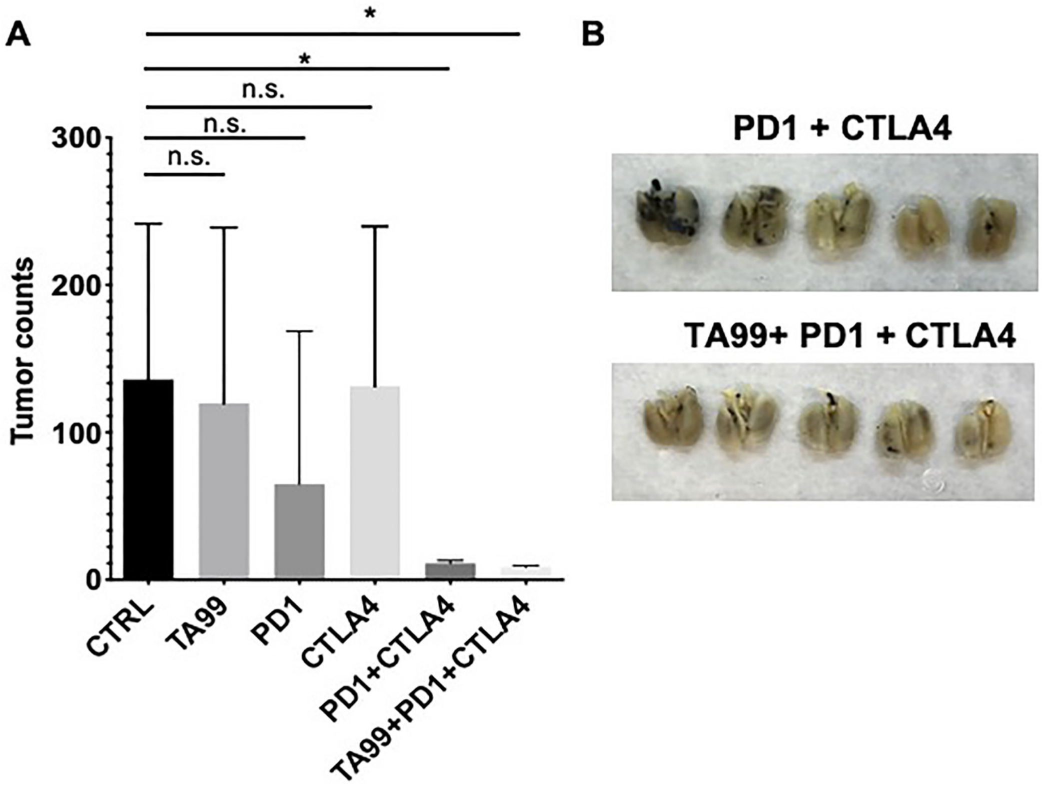

Supplementary Figure 4: Treatment with TA99 does not enhance the efficacy of anti-CTLA4/anti-PD1 combination in the B16 lung metastases model. B16F10 cells were injected via tail vein into C57BL/6 mice which were treated with immune checkpoint blocking antibodies alone or in combination and combined with TA99 anti-tumor antibodies. Number of pigmented nodules in the lungs of individual animals (**A**), and images of lungs from animals treated with the different combination therapies (**B**) are shown. $^{*}p < 0.05$.

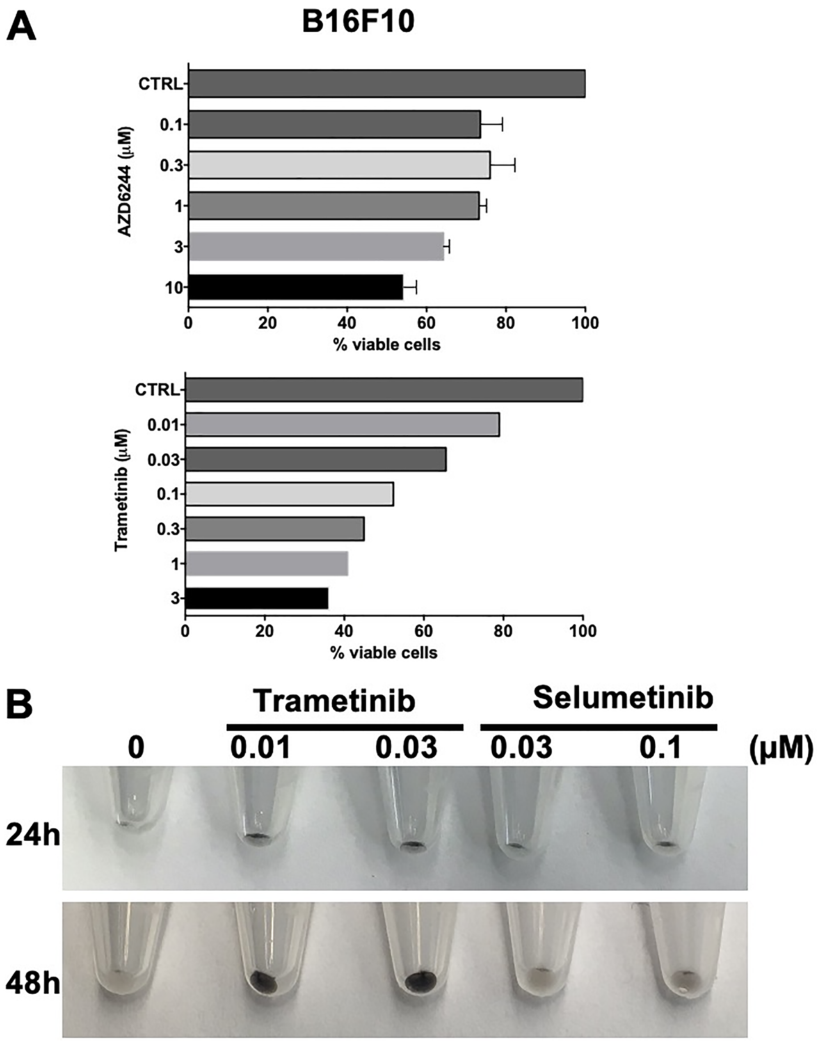

Supplementary Figure 5: MEK inhibitors partially inhibit priliferation and induce pigmentation in B16 melanoma cells. 3-(4,5-dimethylthiazol-2-yl)-5-(3-carboxymethoxyphenyl)-2-(4-sulfophenyl)-2H-tetrazolium (MTS) viability assay shows a MEKi (selumetinib and trametinib) dose-dependent partial inhibition of cell viability in B16 melanoma cells (**A**); Percent of viable cells compared to DMSO control is shown (assay performed in triplicate). In line with previous reports of MEKi-induced increased expression of melanosomal antigens in BRAF mutant cells after 24 and 48 h incubation with MEKi, B16 melanoma cells showed an increase in pigment production as shown after cells were harvested and spun down (**B**).
